# The Forty-First Annual Report of the Commissioners in Lunacy for Scotland

**Published:** 1899-10-21

**Authors:** 


### THE FORTY-FIRST ANNUAL REPORT OF THE COMMISSIONERS IN LUNACY FOR SCOTLAND.

The Commissioners in Lunacy for Scotland have just issued their forty-first annual report. We have now a period of full forty years to look back on, and an enormous mass of statistics to peruse if inclination prompt us in that direction. At the time of the formation of the Lunacy Board, Scotland had 5,824 lunatics, or rather, that was the number of which official cognisance was taken. The number has now swollen to 15,399, that is, it has almost trebled during the forty years, and there is no sign, no hope, that this increase in the registered insane is likely to stop, much less to decrease. The vast majority are pauper lunatics, and we find that the ratio of these to the general population reached its highest mark in 1898, being 301 per 100,000, as against 293, the highest in any previous year. The Commissioners point out that the number of lunatics under jurisdiction of the Board has increased by 164 per cent., whilst the increase of the population has been only 41 per cent. This is not very consoling reading to the careful Scotchman, in whatever way the increase may be accounted for; and the man who has a turn for statistics may carry these figures out to the end of 1940 and show us the number of lunatics which Scotland will have to maintain, and the sum which will have to be paid for them. It may be assumed that each pauper lunatic will cost the country about £25 per annum.

Apart from these sad causes of reflection, it may be said that this Blue Book is, like its predecessors, an eminently readable and satisfactory one, with perhaps one exception, which we shall mention further on, and that it is much less toilsome and wearisome than such things are wont to be. There is a clearness in the arrangement of the letterpress which makes the statistics easy to follow.

We notice that the recovery rate in the royal and district asylums stands at 40 per cent. on the admissions; in private asylums at 41 per cent., and in parochial asylums at 42 per cent. Even the lunatic wards of workhouses show a small percentage of recoveries. The Commissioners most correctly point out that very erroneous inferences might be drawn from such figures, and that it is essential to take into consideration the class of patients and the nature of the cases admitted before drawing any conclusions; and, we might add, that even knowing these a long series of statistics would be necessary.

The percentage of deaths on the number resident was a fraction over eight.

The Commissioners have always evinced much interest in the staff of attendants and nurses in the Scotch asylums, and many of their recommendations, if carried out, would be calculated to decrease the number of resignations. But we doubt whether anything would have such a favourable effect on the stability of the nursing staffs of asylums as a determination on the part of medical superintendents never to engage anyone as attendant or nurse who has served in any other asylum.

The boarding-out of pauper lunatics in private dwellings has been pushed to a much greater extent in Scotland than it has in England. In the former country upwards of 1,000, being about one-thirteenth of the whole, were in this manner provided for during 1898, whereas in England the proportion is so small that it does not appreciably lessen the enormous number of the pauper insane confined in our county asylums.

In all parts of Great Britain the pauper lunatic is nowadays very well taken care of, and so is the private patient who can pay £2 a week and upwards; but for the classes who are not able to pay more than 10s. or 20s. a week, the case is far otherwise, and these unfortunate creatures inevitably drift towards the pauper wards of our asylums. It might be thought that our lunatic hospitals would provide for this much to be pitied class, and to some slight extent they do; but the temptation to admit patients who pay more than 20s. is so great that too often the coffers of the asylum are enriched and the poor patient goes to the wall. The Commissioners think that the District Lunacy Boards should be allowed to build annexes at their asylums for private patients, and it is probable that in some cases advantage would be taken of such a law, but we must confess to a lack of faith in permissive legislation.

There are several points in which the Northern and the Southern Boards take different views. For instance, as regards single-bedded rooms. The English Commissioners think these should be sufficient for one-fourth of the total number of patients in an asylum, while the Scotch do not much believe in these single rooms at all, and the latter are of opinion that the excitement, noise, and faulty habits of a patient are best remedied by placing him in an associated dormitory under suitable attendants, and not by locking him in a single room. Such a system might be most unpleasant for the quieter patients when the breaking-in period was in hand; but when we remember that the abolition of airing-court walls, the boarding-out of pauper lunatics, the open-door system, and other reforms came to us from the north, it is not unlikely that the Scotch Commissioners are right again, and that we have yet another lesson to learn from them.

Consumption is one of the commonest diseases in asylums and out of them, and the Commissioners think that the information relative to its prevalence would be more accurate if post-mortem examinations were made in every case of death, and they add that asylum authorities are not making an adequate use of the powers and duties committed to them if they do not encourage such examinations. Here we are compelled to differ. Surely it is no part of the medical officer's duty to make these examinations, except in such cases as may be directed by the coroner, or as may be required to enable a correct medical certificate of the causes of death to be given. The wholesale making of these post-mortem examinations rides rough-shod over the feelings of the poor; it is quite unnecessary from a scientific standpoint, and we hope it will not be carried out; or, if carried out, that the common law will step in and show that the medical superintendent of an asylum has not charge of a body for the purpose of making a post-mortem examination on it.

During 1898 Scotland got rid of 38 pauper lunatics from its asylums. Of these, 17 were sent to England, and 21 to Ireland. It has always been a mystery to us why the exchange system should not be in vogue here. In our English county asylums, Scotch patients exist by tens and Irish by hundreds, and both these are allowed to remain with us, without so far as we know any attempt being made to remove them.

